# SApredictor: An Expert System for Screening Chemicals Against Structural Alerts

**DOI:** 10.3389/fchem.2022.916614

**Published:** 2022-07-13

**Authors:** Yuqing Hua, Xueyan Cui, Bo Liu, Yinping Shi, Huizhu Guo, Ruiqiu Zhang, Xiao Li

**Affiliations:** ^1^ Department of Clinical Pharmacy, The First Affiliated Hospital of Shandong First Medical University and Shandong Provincial Qianfoshan Hospital, Shandong Engineering and Technology Research Center for Pediatric Drug Development, Shandong Medicine and Health Key Laboratory of Clinical Pharmacy, Jinan, China; ^2^ Institute of Materia Medica, Shandong First Medical University & Shandong Academy of Medical Sciences, Jinan, China; ^3^ Department of Clinical Pharmacy, Shandong Provincial Qianfoshan Hospital, Shandong University, Jinan, China

**Keywords:** SApredictor, structural alerts, web-server, toxicity prediction, expert system

## Abstract

The rapid and accurate evaluation of chemical toxicity is of great significance for estimation of chemical safety. In the past decades, a great number of excellent computational models have been developed for chemical toxicity prediction. But most machine learning models tend to be “black box”, which bring about poor interpretability. In the present study, we focused on the identification and collection of structural alerts (SAs) responsible for a series of important toxicity endpoints. Then, we carried out effective storage of these structural alerts and developed a web-server named SApredictor (www.sapredictor.cn) for screening chemicals against structural alerts. People can quickly estimate the toxicity of chemicals with SApredictor, and the specific key substructures which cause the chemical toxicity will be intuitively displayed to provide valuable information for the structural optimization by medicinal chemists.

## Introduction

Nowadays, the development of chemical toxicology studies has provided us with extensive compound toxicity data. By analyzing and mining the existing toxicological experimental data, computational models can be established to predict the toxicity of chemical compounds around our lives. Compared with biological experimental methods, the computational methods were always green, fast, cheap, and accurate (Yang et al., 2018c). More importantly, toxicity can be predicted with computational models even before a chemical is synthesized or isolated. In the past decades, several expert systems, for example, Toxtree (Patlewicz et al., 2008) and OECD QSAR Toolbox (https://qsartoolbox.org/etc), and web-servers, for example, admetSAR (Yang et al., 2018a), ToxAlerts (Sushko et al., 2012), ADMETlab (Xiong et al., 2021), pkCSM (Pires et al., 2015), and vNN (Schyman et al., 2017) have also been proposed for *in silico* toxicity estimation.

The quantitative structure activity relationships (QSAR) method is one of the most widely used computational approaches for toxicity prediction, and many QSAR models are reported every year. However, these QSAR models based on machine learning methods tend to be “black box” models, which have limited the application in the prediction of various properties for regulatory agencies (Alves et al., 2016). In addition, the machine learning models need to face the problem of applicability domains (ADs) definition. Defining ADs are essential for regulatory acceptance of QSAR models, but there is less standard definition of AD for the global QSAR model nowadays, and many published QSAR models do not provide ADs (Wang et al., 2021).

Structural alert (SA) is another widely accepted tool for toxicity prediction in recent years, which can be defined as the key substructure which can cause specific toxicity. SA has been commonly used for assessment of many toxicity endpoints (Benigni et al., 2013; Li et al., 2017a; Limban et al., 2018; Kalgutkar, 2020; Cui et al., 2021; Huang et al., 2021; Shi et al., 2022) since Ashby and Tennant (1988) proposed the concept in 1985. The SAs can visually alert the toxicity of chemicals by displaying the key fragments responsible for drug toxicity because of the direct derivation from mechanistic knowledge. Therefore, SAs can provide valuable guidance and reference for structural optimization by medicinal chemists to reduce the risk (Yang et al., 2018c).

In the present study, we focused on screening chemicals against structural alerts, including 1) the identification of specific SAs responsible for the toxicity endpoints most concerned in drug studies based on a database with high quality toxicity data and the collection of reported SAs from research publications; and 2) the development of web-server for screening chemicals against structural alerts.

## Materials and Methods

### Data Collection and Preparation

The data for identification of structural alerts were collected from 1) the databases such as ChEMBL (Gaulton et al., 2011), ChemIDplus (Tomasulo, 2002), Comparative Toxicogenomics Database (CTD) (Davis et al., 2018), Carcinogenic Potency Database (CPDB) (Gold et al., 1984) and DrugBank (Wishart et al., 2017) and 2) peer-reviewed publications through manually filtering and processing. We focused on 22 toxicity endpoints which are of most concern in environmental toxicology and drug discovery, including acute oral toxicity (Li et al., 2014), chemical aquatic toxicity [*Tetrahymena pyriformis* (Cheng et al., 2011), *Daphnia magna* (Gajewicz-Skretna et al., 2021), and fathead minnow (Sun et al., 2015)], chemical-induced hematotoxicity (Hua et al., 2021), drug-induced neurotoxicity (Jiang et al., 2020), drug-induced autoimmune diseases (Wu et al., 2021), drug-induced ototoxicity (Huang et al., 2021), drug-induced rhabdomyolysis (Cui et al., 2019), endocrine disruption (Chen et al., 2014), eye irritation (Wang et al., 2017), hepatotoxicity (Li et al., 2018), hERG inhibition (Li et al., 2017c), honey bee toxicity (Li et al., 2017b), inhalation toxicity (Cui et al., 2021), mitochondrial toxicity (Nelms et al., 2015), mutagenicity (Yang et al., 2017), nephrotoxicity (Shi et al., 2022), non-genotoxic carcinogenicity (Benigni et al., 2013), reproductive and development toxicity (Fan et al., 2018; Jiang et al., 2019), skin sensitization (Di et al., 2019), and toxicity on avian species (Zhang et al., 2015). For each toxicity endpoint, we searched the literature separately and included the publications with the same definition of the toxicity endpoint and consistent toxic/non-toxic classification criteria.

The datasets were prepared in following steps to guarantee the quality and reliability of the data: 1) removing mixtures, inorganic, salts, and organic metallic compounds; 2) removing compounds without explicit description for toxicity properties or have inconsistent results in different experimental groups; 3) removing the fuzzy, uncertain, and obviously uncorrected data points; and 4) standardization and representing as canonical SMILES (O’Boyle, 2012).

### Identification of Structural Alerts

**FIGURE 1:**
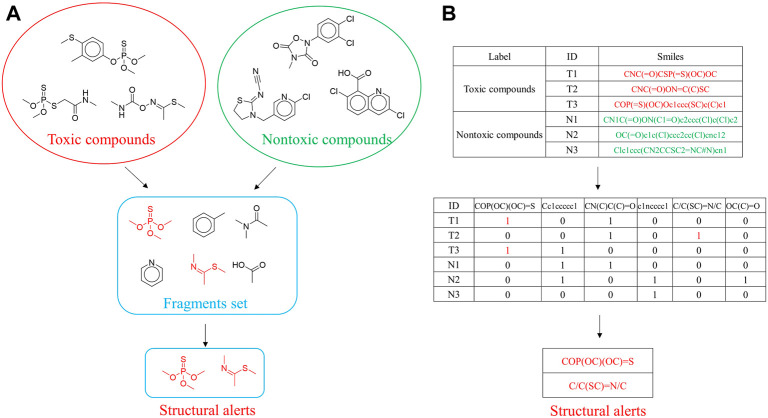
SARpy and fingerprints filter approaches implemented for structural alerts identification. **(A)** SARpy method is a fragment-based method, which can cut all possible bonds to obtain substructures. **(B)** Fingerprints filter approach would regard the predefined substructures as potential structural alerts.

The structural alerts were identified with two different methods, including SARpy (Ferrari et al., 2013) and fingerprints filter (Yang et al., 2020). Both the methods were based on frequency analysis, the general idea of which was to find some substructures presented more frequently in toxic compounds than in non-toxic ones (Yang et al., 2020). If a substructure presented far more frequently in toxic compounds than non-toxic compounds, the presence of such a substructure could alert to toxicity. Thus, this substructure should be regarded as a structural alert responsible for the specific toxicity. The flow of these two methods for identifying structural alerts was shown in Figure 1. SARpy is a python-based standalone software program for automated QSAR modeling. This program has been well-described in detail by Ferrari et al. (2013). Using SMILES-based algorithms, SARpy can cleave the compounds to obtain all possible fragments, and the potential structural alerts can be obtained by frequency analysis. In this study, rule sets were generated using standard settings; the substructures are composed of minimum two and maximum 18 atoms and occurring in a minimum of three substances. For the fingerprints filter method, the well-defined fingerprints of various lengths were utilized as the source of substructures. In the present study, the structural alerts were identified with a f-score and positive rate of each substructure from Klekota-Roth fingerprint (KRFP) calculated with a PaDEL-Descriptor (Yap, 2011), which contained 4,860 predefined structural fragments. The positive rate (PR) of a substructure is defined as Eq. 1:

$$PR=\frac{N_{fragment\_positive}}{N_{fragment}}.$$ (1)

where N_*fragment_positive*_ is the number of toxic compounds containing the fragment, and N_*fragment*_ is the total number of compounds containing the fragment. For each specific endpoint, only the fragments presented in six or more compounds were maintained. The fragments with f-score ≥0.005 and positive rate ≥0.65 were identified as structural alerts.

The structural alerts were converted into SMARTS patterns (Hanson, 2016) and stored in the MySQL database. The SMARTS pattern is a language that allows users to specify substructures using the rules, which are straightforward extensions of SMILES. With SMARTS, flexible, and efficient substructure-search specifications can be made in terms that are meaningful to chemists, a compound can be matched against the alert in an automatic manner using one of the available chemical libraries (Sushko et al., 2012).

In addition, we also collected the structural alerts reported in the peer-reviewed publications. The collected structural alerts were also converted into SMARTS patterns, and the duplicates were removed.

### Applicability Domain Definition

As emphasized by OECD principles, a well-defined applicability domain (AD) was preferred to make models more precise and robust (Yang et al., 2020). From the viewpoint of predictive performance, AD can also be helpful for improving the accuracy of SAs. Numerical relationships between chemical descriptors and toxicity values from training set are the basis of many applicability domain definition techniques, especially for QSAR models. However, AD for SAs have not been defined using these methods since the alerts are always a combination of structural information, toxic or non-toxic testing outcomes, and expert knowledge, which are used to directly link substructures with potential activity (Ellison et al., 2011). To date, there has been no single generally accepted algorithm for determining the AD on SAs. Since no chemical descriptors were used for SA model building, structural similarity could be the most appropriate measure to define the AD. Structural similarity is a popular AD definition method based on the concept that if a query chemical can be defined as similar to the chemicals in the training data, then it can be considered within the AD (Kühne et al., 2006; Ellison et al., 2011). In the present study, the similarity matrix was calculated employing the Tanimoto coefficient (Tc) (Godden et al., 2000; Bajusz et al., 2015) based on the KRFP fingerprint. The Tc is defined as Tc = Nab/(Na+ Nb− Nab), with Na being the number of bits set on in molecule a, Nb is the number of bits set on in molecule b, and Nab is the number of bits set on common to both molecules (Godden et al., 2000). The cutoff similarity value was defined as 0.5, thus if a query compound had a similarity value of ≥0.5 to at least one compound in the training set, it would be considered to be within the AD.

### Toxicity Prediction With Structural Alerts

The structural alerts were assessed with the specific dataset of each endpoint. The compounds were input as SMILES and queried for matching the specific structural alerts with RDKit (Lovrić et al., 2019). If a compound contains one or more structural alerts, it would be predicted to have the specific toxicity. The evaluation was based on the counts of true positives (TP), false positives (FP), true negatives (TN), and false negatives (FN). Several statistical parameters were also calculated, including the total accuracy (Q), sensitivity (SE), specificity (SP), and positive predictive value (PPV). These parameters are calculated with Eqs. 2–5:

$$Q=\frac{TP+TN}{TP+FN+TN+FP},$$ (2)

$$SE=\frac{TP}{TP+FN},$$ (3)

$$SP=\frac{TN}{TN+FP},$$ (4)

$$PPV=\frac{TP}{TP+FP}.$$ (5)

### Web-Server Implementation

The prediction system was developed employing the Python web framework of Django. The system was deployed on an elastic compute service from Huawei Cloud running an Ubuntu Linux system. The web access was enabled *via* the Nginx web-server and the interactions between Django and proxy server were supported by mod_wsgi v3.3. A user-friendly web interface was provided for computational prediction using a cascading style sheet (CSS) and Python script.

## Results and Discussion

### Compound Libraries and Sets of Structural Alerts

In total, more than 35,716 annotated measurements of about 27,500 unique compounds were collected, including thousands of FDA-approved and experimental drugs, pesticides, environmental agents, and industrial chemicals. As shown in Table 1, these chemicals were checked and divided into 22 subsets, according to different toxicity endpoints.

**TABLE 1:** Number of data points and structural alerts in the data set.

Endpoints	Species	Annotated data points			Structural alerts
		Positive	Negative	Total	
Acute oral toxicity	Rat	3,722	2,129	5,851	35
Chemical aquatic toxicity: *Tetrahymena pyriformis*	*Tetrahymena pyriformis*	1,088	350	1,438	110
Chemical aquatic toxicity: *Daphnia magna*	*Daphnia magna*	307	178	485	57
Chemical aquatic toxicity: fathead minnow	Fathead minnow	451	510	961	51
Chemical-induced hematotoxicity	Human	632	1,515	2,147	12
Drug-induced autoimmune diseases	Human	148	450	598	12
Drug-induced neurotoxicity	Human	329	355	684	18
Drug-induced ototoxicity	Human	497	740	1,237	15
Drug-induced rhabdomyolysis	Human	183	1,321	1,504	8
Endocrine disruption	*In vitro* and *in vivo* assays	433	835	1,268	7
Eye irritation	Rabbit	1,874	1,046	2,920	9
Hepatotoxicity	Human and animals	1,338	857	2,195	51
hERG inhibition	*In vitro* assays	1,186	1,148	2,334	24
Honey bee toxicity	Honey bee	74	176	250	7
Inhalation toxicity	Human	136	468	604	81
Mitochondrial toxicity	Human	171	113	284	41
Mutagenicity	Salmonella	3,503	1,709	5,212	809
Nephrotoxicity	Human and animals	287	238	525	117
Non-genotoxic carcinogenicity	Rat	603	460	1,063	129
Reproductive and development toxicity	Rodents	862	961	1,823	20
Skin sensitization	Rodents	370	417	787	121
Toxicity on avian species	Avian species	140	149	289	22
Summary		19,053	16,663	35,716	1,834

Through the identification and literature retrieval, a total of 1,834 structural alerts were identified and collected for the aforementioned 22 toxicity endpoints, as shown in Table 1. ToxAlerts and Toxtree are two popular tools for the estimation of potential adverse reactions of chemicals. ToxAlerts is a web-server of structural alerts, which collected SAs defined by experts or detected by computational tools. The latest ToxAlerts (accessed on 8 April 2022) contains 814 structural alerts for 13 toxicity endpoints, as shown in Supplementary Table S1. Toxtree is another user-friendly open-source application, which is able to estimate toxic hazard by applying a decision tree approach. In the latest version (Toxtree 3.1.0), in addition to the three Cramer Decision Trees (Cramer Rules, Revised Cramer Decision Tree, and Cramer Rules, with Extensions), it contains 499 structural alerts for 13 toxicity endpoints, as shown in Supplementary Table S2. To our knowledge, this may be the largest structural alert database with specific toxicity endpoints until now. In addition, several toxicity endpoints which get a lot of concerns (hepatotoxicity, nephrotoxicity, reproductive and development toxicity, hERG inhibition, hematotoxicity, mitochondrial toxicity, *etc*.) were included in SApredictor while not in ToxAlerts or Toxtree.

### Performance of Toxicity Prediction With Structural Alerts

The performances of the structural alerts on toxicity prediction are shown in Table 2. The results suggested that for most endpoints, the structural alerts can well distinguish toxic compounds from non-toxic ones. For different toxicity endpoints, the disparity was observed in the performance. This can be attributed to that the complexity of the mechanisms of action (MOAs) of different toxicity endpoints vary greatly, and the sizes of the data are also different, which lead to differences in the representativeness of SAs and the ability to distinguish between toxic and non-toxic compounds.

**TABLE 2:** Performance of toxicity prediction with structural alerts.

Endpoints	SE (%)	SP (%)	Q (%)	PR (%)
Acute oral toxicity	66.01	60.69	64.07	74.59
Chemical aquatic toxicity: *Tetrahymena pyriformis*	75.92	90.29	79.42	96.05
Chemical aquatic toxicity: *Daphnia magna*	80.46	65.73	75.05	80.19
Chemical aquatic toxicity: fathead minnow	72.95	75.88	74.51	72.79
Chemical-induced hematotoxicity	11.87	98.09	72.71	72.12
Drug-induced autoimmune diseases	26.35	97.11	79.60	75.00
Drug-induced neurotoxicity	34.65	96.90	66.96	91.20
Drug-induced ototoxicity	21.53	98.11	67.34	88.43
Drug-induced rhabdomyolysis	22.40	98.41	89.16	66.13
Endocrine disruption	21.25	94.49	69.48	66.67
Eye irritation	45.20	64.63	52.16	69.60
Hepatotoxicity	76.38	39.79	62.10	66.45
hERG inhibition	81.79	47.82	65.08	61.82
Honey bee toxicity	75.68	92.61	87.60	81.16
Inhalation toxicity	89.71	81.41	83.28	58.37
Mitochondrial toxicity	32.16	87.61	54.23	79.71
Mutagenicity	97.52	43.30	79.74	77.90
Nephrotoxicity	85.37	47.48	68.19	66.22
Non-genotoxic carcinogenicity	60.53	55.43	58.33	64.04
Reproductive and development toxicity	24.71	89.39	58.80	67.62
Skin sensitization	79.46	50.36	64.04	58.68
Toxicity on avian species	73.57	46.98	59.86	56.59

It was worth pointing out that compared with QSAR models, the prediction accuracy of structural alerts did not have any advantage in most cases. However, different from the QSAR’s black box model, the structural alerts can visually display the fragments that lead to specific toxicity of compounds, which is conducive to the targeted optimization of toxic structures and the study of toxic mechanisms (Yang et al., 2018c; Shi et al., 2022).

To ensure usefulness of the prediction system, it will be updated regularly with additional structural alerts based on available data, whether identified by ourselves or reported by peer-reviewed publications. If high quality datasets with new endpoints are reported, new structural alerts will be identified and implemented in our database.

### Web Interface and Usage of the Structural Alerts

**FIGURE 2:**
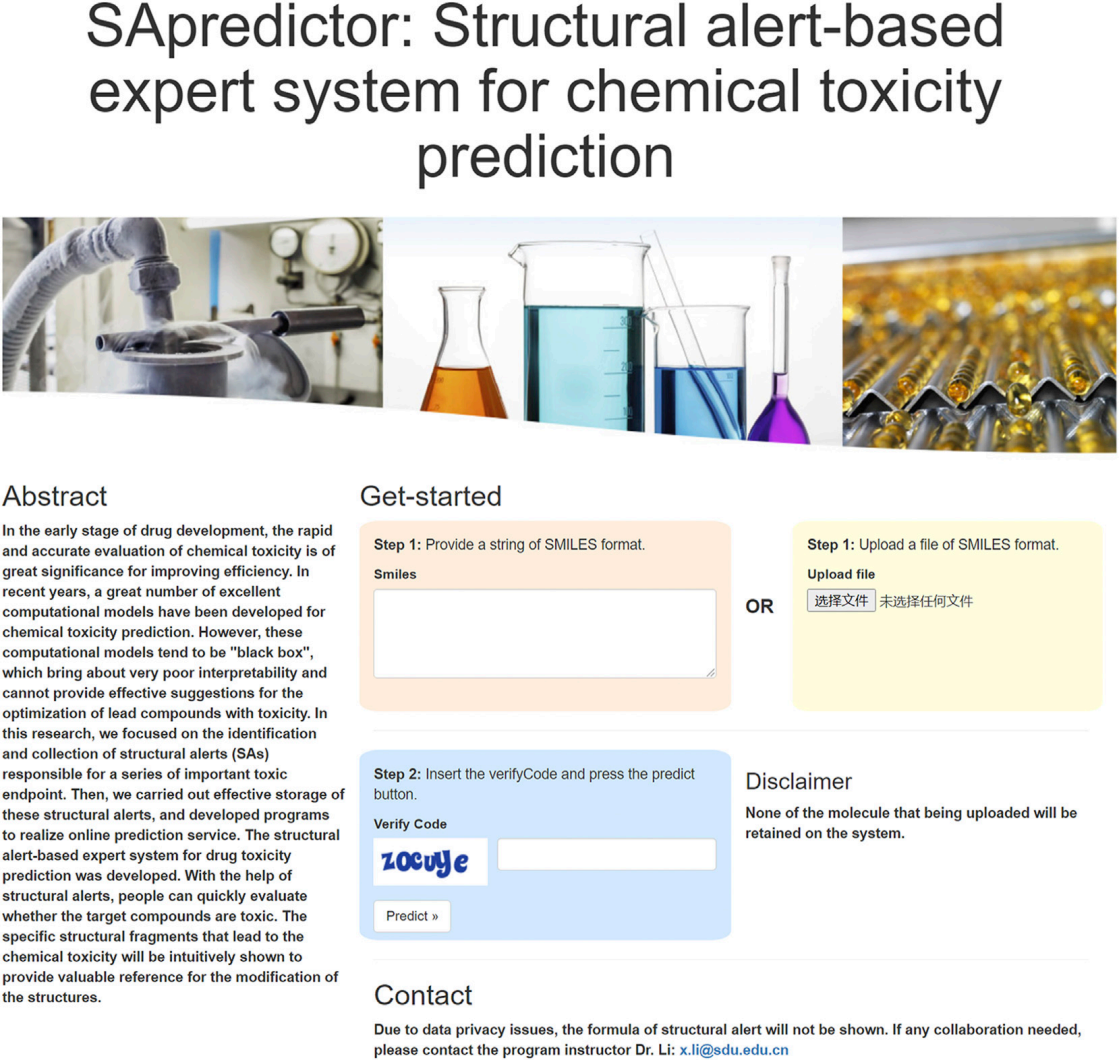
SApredictor main page. From this page, users can submit the query structure.

Based on distributed storage architectures, a piece of software for the estimation of chemical toxicity with structural alert was developed. The software provides a user-friendly interface *via* www.sapredictor.cn. A screenshot of the web-server is shown in Figure 2. Users can submit compound structures in two different ways: 1) enter the SMILES of small compounds in the dialog box; 2) click the “Select File” button to upload the structure file of compounds with SMILES format. After entering the verification code, users can click the “Predict” button to complete the task submission.

**FIGURE 3:**
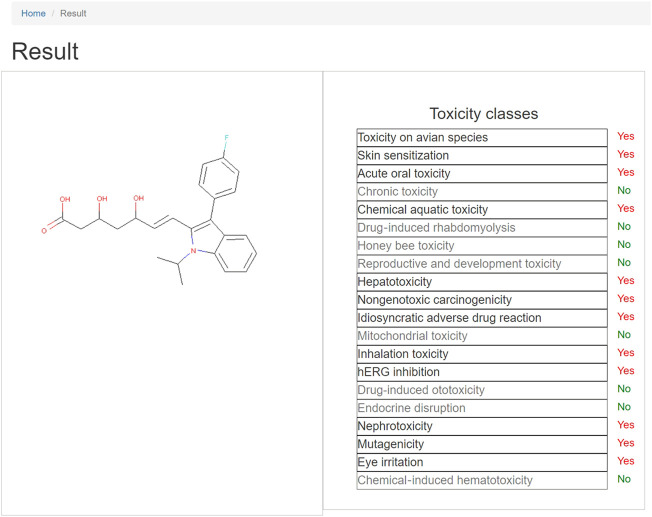
Structural alert-based toxicity predictions result page. When clicking the name of the toxicity endpoint withe positive result, a drop-down list will appear with the ID of structural alerts. Users can click the ID, and then the fragments of the compound will be highlighted in red, so that researchers can view the specific substructure that causes the toxicity of the compound.

After the matching of the structural alerts, it will be redirected to the results page, as shown in Figure 3. On the left is the 2D structure of the query compound and on the right is the toxicity endpoints and corresponding predicted result. Where “Yes” indicates that the query structure contains one or more structural alerts of the specific toxic property, that is, the compound has the potential of the specific toxicity, while “No” indicates the query compound does not have the potential of the specific toxicity. For the toxicity endpoint with the result of “Yes,” click the name of the toxicity endpoint and a drop-down list will appear listing the ID of structural alerts. When clicking the ID, the fragments of the compound will be highlighted in red, and the SMARTS of the alert will also be available on the page. The researchers can view the specific substructure that causes the toxicity of the compound.

## Conclusion and Perspectives

In summary, we have described here a web-server, named SApredictor, for screening chemicals against structural alerts *via* www.sapredictor.cn. In SApredictor, 1,834 structural alerts for 22 different toxicity endpoints were extracted from more than 35,716 toxicity annotated data points or collected from peer-reviewed publications. Users can quickly estimate the toxicity of compounds and visually display the fragments, which contribute to their toxicity. The web-server will never retain any information submitted to it because of the confidentiality of users’ projects. We hope that the software should facilitate the process of drug discovery and development by enabling the rapid and rational screening, design, evaluation, and prioritization of drug candidates.

It is worth pointing out that use of structural alerts alone may suffer from false positives, such as skin sensitization and toxicity on avian species in the present study. The structural alerts were always identified by statistics-based methods or knowledge of toxic mechanisms, which would make them be overtly common and lead to many non-toxic structures being estimated as toxic. On the other hand, it is debatable whether compounds which do not contain any SA can be classified as non-toxic. Toxicity prediction based on SA is based on the existing knowledge. The compounds with SAs are always toxic, but whether those without SA are non-toxic needs more toxicity data support. Yang et al. (2018b) proposed a concept of non-toxic substructures, whose appearance will reduce the probability of a compound becoming toxic (Yang et al., 2018b). In Wang et al. (2012) work, modulating factors that suppressed the toxic effects of SAs were extracted and practice on carcinogens (Wang et al., 2012). Non-toxic substructures and modulating factors could be beneficial supplements to SAs. In addition to optimizing the identification method of structural alerts, defining the applicability domain for the structural alerts in a reasonable strategy may be helpful to improve the predictive performance and eliminate the worries. We will continue to work in both directions to improve the predictive ability of structural alerts and make them more useful.

## Data Availability

The original contributions presented in the study are included in the article/Supplementary Material; further inquiries can be directed to the corresponding authors.
