# Supplementary material for: SApredictor: An Expert System for Screening Chemicals Against Structural Alerts
Source: Front Chem. 2022 Jul 13;10:916614. doi: 10.3389/fchem.2022.916614 (PMC9326022; doi:10.3389/fchem.2022.916614)
Supplement: Supplementary file 1 [file DataSheet1.docx]

Table S1. The number of endpoints and structural alerts in ToxAlerts

| Endpoints | Structural alerts |
| --- | --- |
| Melting Point | 1 |
| Acute Aquatic Toxicity | 2 |
| Custom filters | 2 |
| UNIFAC | 55 |
| LD50_mo_oral | 20 |
| SMARTCyp | 41 |
| Glaxo Wellcome | 124 |
| Dundee | 105 |
| MLSMR | 115 |
| LINT | 57 |
| Inpharmatica | 91 |
| SureChEMBL | 166 |
| Endocrine disruption | 35 |

Table S2. The number of endpoints and structural alerts in Toxtree

| Endpoints | Structural alerts |
| --- | --- |
| Cramer rules | 36 |
| Revised Cramer Decision Tree | 34 |
| Kroes TTC decision | 41 |
| Verhaar scheme | 33 |
| Carcinogenicity (genotox or nongenotox) and mutagenicity rulebase by ISS | 63 |
| In vitro mutagenicity (Ames test) alerts by ISS | 51 |
| Skin irritation/skin corrosion | 70 |
| Eye irritation/corrosion | 63 |
| Skin sensitisation reactivity domains | 5 |
| START biodegradability | 32 |
| Cytochrome P450-Mediated Drug Metabolism | 4 |
| Structure Alerts for the in vivo micronucleus assay in rodents | 37 |
| Structural Alerts for Functional Group Identification | 90 |
| Protein binding alerts | 5 |
| DNA binding alerts | 5 |
| Cramer rules, with extensions | 42 |
